# Detection of Pseudorabies in Dogs in Slovenia between 2006 and 2020: From Clinical and Diagnostic Features to Molecular Epidemiology

**DOI:** 10.1155/2023/4497806

**Published:** 2023-04-15

**Authors:** Danijela Černe, Peter Hostnik, Ivan Toplak, Polona Juntes, Tomislav Paller, Urška Kuhar

**Affiliations:** ^1^Institute of Microbiology and Parasitology, Virology Unit, Veterinary Faculty, University of Ljubljana, Slovenia; ^2^Institute of Pathology, Wild Animals, Fish and Bees, Veterinary Faculty, University of Ljubljana, Slovenia; ^3^National Veterinary Institute, Veterinary Faculty, University of Ljubljana, Slovenia

## Abstract

Pseudorabies (PR) is one of the most economically important diseases in domestic pigs. Since 2010, Slovenia has been free of PR in the domestic pig population, but the disease is endemic in the wild boar population, which can pose a real threat to domestic pigs and other animal species, including dogs. Between 2006 and 2020, infections with the PR virus (PRV) were reported in two pets and three hunting dogs from Slovenia that were found to have a direct contact with the wild boar or raw wild boar or pork meat. Typical clinical signs of PRV infection, including characteristic facial itching, cytopathic effect in cell cultures, positive immunocytochemistry, and positive PCR results confirmed the presence of PRV in all five cases investigated. A phylogenetic comparison of the partial glycoprotein C (gC) genomic region revealed that the Slovenian PRV isolates belong to clade A, with 95.78–100% nucleotide identity with strains isolated from dogs, domestic pigs, and wild boars from Europe. Within phylogenetic comparison of the partial glycoprotein D (gD) and partial glycoprotein E (gE) genomic regions of Slovenian PRV isolates, 100% and 99.12%–100% nucleotide identities were detected, respectively, suggesting low diversity between the PRV strains identified in dogs in Slovenia. This study provides the first molecular characterization of PRV in dogs and suggests that similar PRV strains circulate in the wild boar populations in this geographic area.

## 1. Introduction

Pseudorabies (PR), also known as Aujeszky's disease (AD), is one of the most economically important diseases of domestic swine and is caused by Suid-alphaherpesvirus 1 (SuHV-1), a member of the genus *Varicellovirus*, family *Herpesviridae*, and subfamily *Alphaherpesvirinae* [1]. Pseudorabies virus (PRV) is an enveloped double-stranded DNA virus with a genome of 143°kb that encodes 70 different proteins [2]. The viral genome consists of a unique long (UL) region, internal repeat sequences (IRS), a unique short (US) region, and terminal repeat sequences (TRS) [3]. For PRV, 11 glycoproteins (gB, gC, gD, gE, gG, gH, gI, gK, gL, gM, and gN) have already been characterized in the lipoprotein membrane alone [3, 4]. One of the most variable regions of the genome is glycoprotein C (gC), which is a part of the UL44 gene and is one of the most important targets for the host immune system [3]. According to characterization by digesting the full genome of PRV by the enzyme BamHI (BamHI-RFLP) [5], field isolates can be classified into four genotypes: genotype I with strains widely distributed in Europe, the Americas, and parts of China [6, 7], genotype II with strains mainly originating from Asian countries [8, 9] and Europe [10, 11], and genotypes III and IV with strains from Denmark and Thailand [12].

PR is a highly contagious viral disease that affects several animal species, including domestic pigs and wild boars as natural hosts of the virus. Pigs infected with PRV can be a source of infection for a wide range of hosts, including cattle, sheep, goats, foxes, mink, wolves, lynxes, dogs, cats, bats, and bears [3, 13]. Species other than suids are considered dead-end hosts because they die before shedding the virus [14]. Nonnatural hosts become infected after direct or indirect contact with the natural host, with the most common direct route of transmission being ingestion of the raw wild boar or pork meat or offal [1]. In nonnatural hosts, typical PRV infection results in severe neurological disease, often accompanied by localized pruritus, and is fatal within a few hours of the onset of the first clinical sign [15, 16]. The localization of pruritus is related to the route of PRV penetration and is defined as a neuropathic form of pruritus or neuropathic pruritus caused by PRV-induced lesions in the peripheral and central nervous systems in combination with an inflammatory response to PRV [13].

PR is a notifiable disease within the population of domestic pigs, and the most effective method for eliminating PR from pigs is large-scale vaccination, which has led to PRV eradication in several parts of Europe [17]. Slovenia, with a population of around 253,770 domestic pigs [18] and wild boars (*Sus scrofa*) with a hunting bag of around 19,900 wild boars per year [18], has been free of PR in domestic pigs since 2010 [19] and the vaccination of domestic pig is prohibited. Similar to pig farming, wild boars are common throughout Slovenia; however, the highest densities are found in the southwest of the country [20]. Contact between domestic pig and wild boar populations is prevented in Slovenia, as sexual attraction or food availability could pose a risk for PRV transmission, as observed by other authors [21].

Two studies published in 2005 and 2014 on serological surveys in Slovenian wild boar showed that PRV circulates with a prevalence of 26% to 45.1% and poses a risk to other susceptible hosts [20, 22]. PRV infections in dogs have been previously reported in Europe, the United States, Japan, and China [6, 10, 23–33]. In most cases where PRV has been reported in dogs, the dogs became infected directly by ingestion or indirectly by inhalation, or via small wounds from contact with infected pigs [1]. The risk of PRV transmission is the highest in hunting dogs that are in close contact with the wild boar, especially if the dogs have access to or are fed with raw wild boar meat as a reward for hunting [23]. Commercial attenuated PRV vaccines could also represent a risk for dogs [34].

The aim of this study was to investigate clinical cases of PR in dogs in Slovenia by histopathology, immunohistochemistry, and laboratory diagnosis using virus isolation and PCR. The aim was also to characterize PRV isolates using phylogenetic analysis based on the partial gC, gD, and gE gene sequences.

## 2. Materials and Methods

### 2.1. Materials

**Figure 1:**
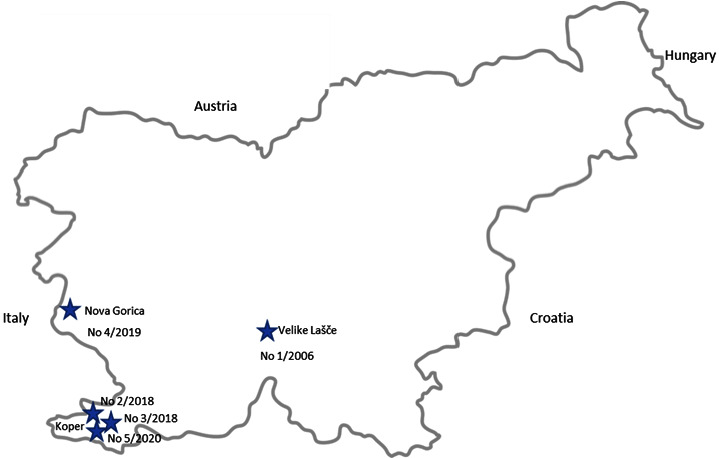
The geographical location of five PRV-positive cases detected in dogs in Slovenia is indicated by stars, including case number and year of the detection.

Five PR-suspected cases in dogs were reported by small animal veterinary practices between January 2006 and January 2020. Suspicion of infection with PRV was based on the typical clinical signs of pruritus and epizootiologic investigations regarding the feeding of dogs with raw wild boar/pork meat and whether close contact with wild boar occurred. Two dogs were pet dogs and three were hunting dogs of different breeds, ages, and sexes (Table 1), and all the five dogs died within two to three days after the onset of clinical signs or were humanely euthanized, and their carcasses were sent to the Veterinary Faculty/National Veterinary Institute for pathological examination for the confirmation of the disease, where a cold chain was established. All these PR cases were diagnosed during the winter months, the first in January 2006 (No. 1/2006), the next two in November 2018 (No. 2/2018, No. 3/2018), one in December 2019 (No. 4/2019), and the last in January 2020 (No. 5/2020) (Table 1). The geographic locations from which these cases originated are shown in Figure 1.

### 2.2. Methods

#### 2.2.1. Postmortem Examination and Sampling

A postmortem examination was performed at the Institute of Pathology, Wild Animals, Fish, and Bees. At necropsy, tissue samples of the brain (parts of the cerebral cortex, cerebellum, and brain stem) and some other tissues (thoracic spinal cord, lung tissue, heart tissue, kidney tissue) were collected for histopathological examination on paraffin tissue slides routinely stained with haematoxylin-eosin (HE) and for immunohistochemistry (IHC). Immunohistochemical examination of the brain tissue was performed with monoclonal antibodies against PRV (ADV-GE, 2CF2, Ingenasa, Madrid, Spain, working dilution 1 : 100) on formalin-fixed paraffin tissue slides using Dako Real EnVision as the detection system and 3,3′-diaminobenzidine tetrachloride (DAB) as the substrate. For the IHC negative control, paraffin slide duplicates were incubated without monoclonal antibodies against PRV. For IHC, slides were deparaffinized and rehydrated, then pretreated in the microwave with citrate buffer pH 6.1 for 10 minutes, and incubated with an anti-PRV antibody for one hour at room temperature. Nonspecific background staining was prevented by incubating the slides in 3% hydrogen peroxide in the wash buffer. Haematoxylin was used as a counterstain. In case No. 1/2006, IHC was also performed on a thoracic spinal cord sample.

Immediately after a postmortem examination and sampling, unfixed brain tissue samples from all cases were sent to the Institute of Microbiology and Parasitology, Virology Unit for confirmation by virus isolation in cell culture, molecular diagnosis, and characterization.

#### 2.2.2. Real-Time PCR

For laboratory detection of PRV by the real-time PCR method, 10% brain tissue homogenates were prepared from the suspect material. Total viral DNA was extracted from the prepared tissue homogenates using the DNeasy Blood and Tissue Kit (Qiagen, Hilden, Germany) according to the manufacturer's instructions. The extracted DNA was tested by a triplex real-time PCR method for specific detection of the PRV and internal control. Two specific primer pairs and two probes targeting the highly conserved sequences within glycoprotein B (gB) and glycoprotein E (gE) regions [35] were performed using the QuantiNova Pathogen + IC kit (Qiagen, Hilden, Germany) in a total reaction volume of 20 *µ*l. For a single reaction, 4 *µ*l of RNase-free water, 5 *µ*l of QuantiNova MasterMix, 2 *µ*l of the 10x IC probe assay, 1 *µ*l of QuantiNova IC DNA (diluted 1 : 100), 1 *µ*l of each primer (SUHV1-dgE-689F, SUHV1-dgE-781R, PrV-gB-778F, and PrV-gB-856R) at a concentration of 20 pmol/reaction, and 0.5 *µ*l of each probe (SUHV1-dgE-723FAM and PrV-gB-809-Cy5) at a concentration of 5 pmol/reaction were combined as a master mix. Finally, 3 *µ*l of DNA template was added and real-time PCR was performed using MX3005P (Agilent, Stratagene, USA). The following thermal profile was used: PCR initial activation step at 95°C for 2 min; 45 cycles with two steps consisting of denaturation at 95°C for 15 s and annealing at 60°C for 1 min.

#### 2.2.3. Virus Isolation on Cell Culture

Virus isolation on cell culture was performed for each sample immediately upon arrival at the Virology Unit, where the cold chain was established. For virus isolation on cell cultures, brain tissue homogenates from all 5 cases (No. 1/2006, No. 2/2018, No. 3/2018, No. 4/2019, and No. 5/2020) prepared for PCR were filtered through disposable filters with an average pore diameter of 0.45 *µ*m (Sartorius, Germany) and inoculated onto a monolayer of porcine kidney 15 (PK -15, ATCC CCL -33) cells. The growth medium for PK-15 consisted of Eagle's Minimum Essential Medium (EMEM, ATCC 30–2003) with 10% FBS (Gibco, Thermo Fischer, USA) and 1% antibiotic and antimycotic (Gibco, Thermo Fischer, USA). After incubation at 37°C in an atmosphere of 5% CO_2_ for 72 hours, the presence of a cytopathic effect (CPE) was assessed daily [36]. For all five cases, virus identity was confirmed by immunocytochemistry on acetone-fixed cell cultures using a similar immunostaining protocol as for paraffin tissue slides. Cell culture slides inoculated with isolates from cases No. 1/2006, No. 2/2018, No. 3/2018, No. 4/2019, and No. 5/2020 were fixed for 10 minutes in acetone, then washed in wash buffer, incubated with monoclonal antibodies against PRV (ADV-GE, 2CF2, Ingenasa, Madrid, Spain, working dilution 1 : 100) for 1 hour, and washed and incubated in 3% hydrogen peroxide in wash buffer for 30 minutes to prevent nonspecific background staining. The reaction was visualized using the Dako Real EnVision detection system and 3,3′-diaminobenzidine tetrachloride (DAB) as the substrate. Haematoxylin was used as a counterstain.

PRV isolates were then subjected to DNA extraction and real-time PCR to confirm the successful isolation of the virus in cell culture. The virus isolation process was continued for 3 passages and virus titration was performed for all five PRV isolates, by the method of Kärber and the titer was expressed per ml [36].

#### 2.2.4. Sequencing, Genetic Characterization, and Phylogenetic Analysis

For sequencing and genetic characterization of PRV isolates, conventional PCR was performed for the five PRV isolates (1^st^ passages) targeting the partial glycoprotein C (gC), partial glycoprotein D (gD), and the partial glycoprotein E (gE). For the amplification of about 800 nt of the gC region, primer pair of gC-For and gC-Rev was used as previously described [6], for the amplification of about 217 nt of the gD region, primer pair FgDm and RgDm was used as previously described [37] and for the amplification of about 493 nt of the gE, primer pair gE-nF and gE-nR was used as previously described [38] (Table 2). For DNA amplification, a platinum TaqDNA polymerase Kit (Invitrogen, Carlsbad, USA) was used in a total volume of 25 *µ*l. For a single reaction, 17 *µ*l of RNase-free water, 2.5 *µ*l of 10X PCR buffer, 0.75 *µ*l of 50 nM MgCl_2_, 0.5 *µ*l of 10 Mm dNTP mix, 0.25 *µ*l of Platinum Taq DNA Polymerase (5 U/*µ*l), 0.5 *µ*l of each primer at a concentration of 20 pmol/reaction as master mix, and 3 *µ*l of DNA template was added. PCR was performed using a Biometra thermocycler (Analytic Jena, Germany). The following thermal profile was used: PCR initial denaturation step at 95°C for 3 minutes and at 72°C for 2 minutes; 35 cycles with three steps consisting of denaturation at 95°C for 30 seconds, annealing for 45 seconds at 62°C for gC, 60°C for gD and gE, and elongation at 72°C for 30 seconds; with a final elongation step at 72°C for 4 minutes and indefinite incubation at 4°C. The result of the amplified isolates was visible after agarose gel electrophoresis (Biometra, Analytic Jena, Germany).

The amplified products were subjected to direct Sanger sequencing using the same primer pairs as for the PCR (Table 2). Sequencing was performed by a commercial sequencing company, Macrogen (Amsterdam, The Netherlands). Nucleotide sequences were assembled using Geneious Prime software (version 2021.2.2.) and compared with other PRV nucleotide sequences from the GenBank database. The nucleotide sequences and the deduced amino acid sequences were aligned using the ClustalW method [39]. Based on the alignment, the nucleotide identities between the Slovenian strains and other strains from the GenBank database were determined. Phylogenetic analysis was performed with the program MEGA version X [40] using the neighbor-joining method with *p*-distance and 1000 bootstrap repeats. The nucleotide sequences of the partial gC, gD, and gE genes for the five PRV isolates identified in this study were deposited in the GenBank database with accession numbers ON025041-45 and OQ242385-94.

## 3. Results

### 3.1. Epizootiologic Investigations, Necropsy, Histopathology, and Immunohistochemistry

Epizootiologic investigations revealed that three cases (No. 2/2018, No. 3/2018, and No. 5/2020) had a direct contact with wild boar during hunting, and other two cases had a contact by consuming raw pork meat (case No. 1/2006) or wild boar meat (case No. 4/2019).

The major lesions noted on necropsy and histopathology are shown in Table 1. The necropsy findings of the first case, No. 1/2006, have been described previously [41]. No necropsy data are available for the two cases diagnosed in 2018 (No. 2/2018, No. 3/2018).

**Figure 2:**
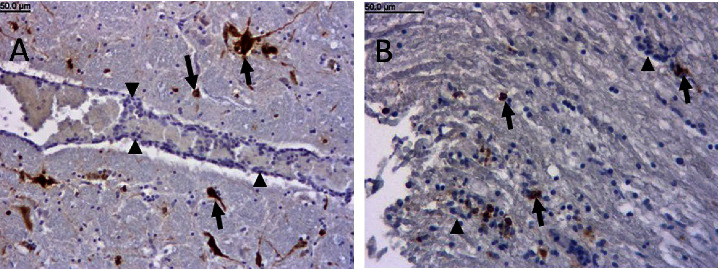
(a) Dog, case No. 4/2019, medulla oblongata, IHC for PRV. Strong positive immunostaining for PRV in neurons and glial cells (brown reaction product with DAB, arrows). Mononuclear inflammatory cells around the blood vessels and endothelial cells are negative for PRV (arrowheads), ×100. (b) Dog, case No. 1/2006, medulla oblongata, IHC for PRV. A mixture of glial and inflammatory cells in the root of the trigeminal nerve (arrowheads) and a small number of cells were positive for PRV (arrows), ×200.

IHC results are shown in Table 1 and Figure 2. The strongest immunostaining was found in the medulla oblongata (Figure 2), in the nucleus of the spinal tract of the trigeminal nerve, and was more intense on one side; however, the IHC-positive cells were distributed in all parts of the medulla, in the grey and white matter, often in clusters. In the brainstem, a similar IHC staining pattern was found in all five cases, but with different intensities. In case No. 1/2006, we also found IHC reactivity in the thoracic segments of the spinal cord. Immunoreactive cells were found in the cranial thoracic segments, where they were the most prominent in the dorsal horns, and the staining was much stronger on one side of the spinal cord. In addition, disseminated IHC-positive cells were found in the ventral horns and in the white matter of the cranial thoracic segments before the lesions and immunopositivity gradually disappeared toward the middle thoracic segments. IHC examination of the brain of case No. 5/2020 revealed a small number of strongly positive neurons and glial cells in the caudal medulla and confirmed PRV infection.

### 3.2. Laboratory Detection of PRV by Real-Time PCR and Virus Isolation in Cell Culture

**Figure 3:**
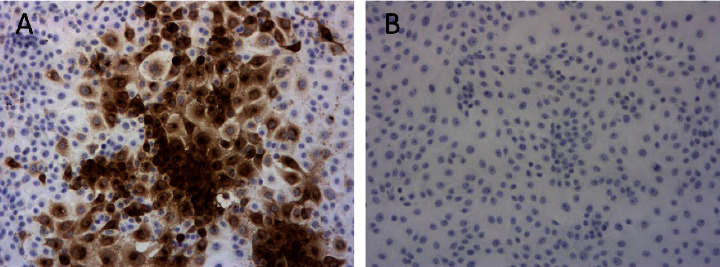
Confirmation of virus isolation on PK-15 cell line by immunocytochemistry. The detected virus on PK-15 (1^st^ passage) with brown reaction in PRV positive sample from case No. 4/2020, ×100, (a) and cell culture of PK-15, negative control, ×100 (b)

Real-time PCR results for the original brain tissue homogenates from four dogs (cases No. 2/2018, No. 3/2018, No. 4/2019, and No. 5/2020) were positive with cycle quantification (*Cq*) values ranging from 29.33 to 33.91 for PRV gE region detection and 29.34 to 34.59 for PRV gB region detection. The original sample from case No. 1/2006 was not tested by the real-time PCR method, so only the result of the cell culture isolate of this PRV strain was included in the subsequent analysis (Table 3). PRVs were successfully isolated from tissue homogenates in a PK-15 cell line from all five real-time PCR PRV-positive dog samples. The presence of typical herpesvirus CPE, including lysis and syncytium formation, was detected in all five samples in all three passages between 24 and 72 hours after inoculation. Virus identity was confirmed by immunocytochemistry (Figure 3) for all five cases. Virus titration of the Slovenian PRV isolates revealed virus titers ranging from 3.1 × 10^7^/ml to 5.6 × 10^8^/ml (Table 3).

After successful PRV isolation and CPE detection, the supernatants from 3 passages of all five PRV isolates were positive in real-time PCR with *Cq* values ranging from 11.29 to 24.35 for the PRV-gE region and from 11.77 to 24.03 for the detection of the PRV-gB region, confirming virus replication in cell culture in all five detected PRV cases (Table 3).

### 3.3. Sequence Comparison and Phylogenetic Analyses

**Figure 4:**
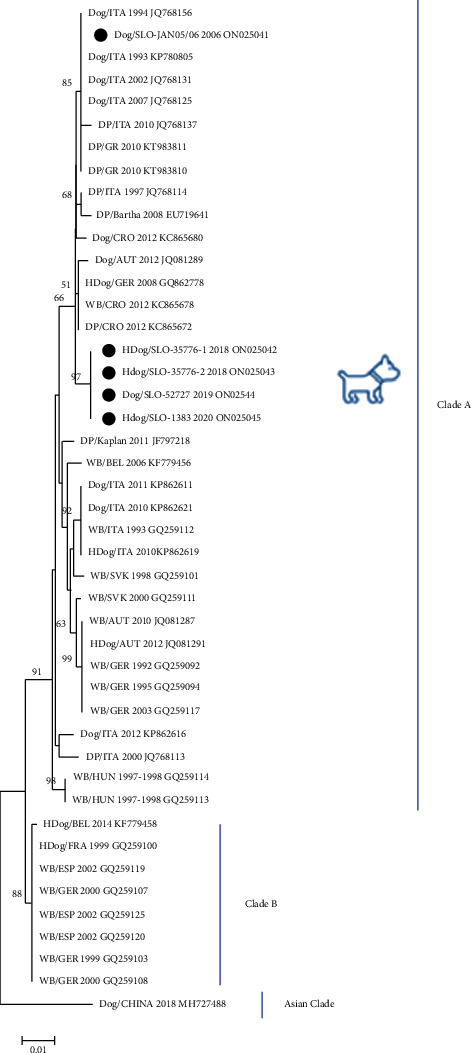
The phylogenetic comparison of the nucleotide sequences of partial gC coding region (genome positions from 53.019 to 53.681, according to reference strain Kaplan JF797218) of the five Slovenian PRV strains isolated from dogs and the 40 PRV sequences from wild boars, domestic pigs, and dogs from Europe and China. The tree was constructed by using the neighbour-joining method with *p*-distance and 1000 bootstrap replicates in MEGA version X [40]. For each strain species, country, the year of isolation, and accession number are reported (WB: wild boar, DP: domestic pig, and HDog: hunting dog). Slovenian PRV strains from this study are indicated with a black dot.

For genetic characterization of the PRV isolates, the partial nucleotide sequences of the gC, gD, and gE genes were successfully obtained by direct sequencing of the PCR products. Alignment of the nucleotide sequences was performed to determine the nucleotide identities between PRV strains isolated from dogs from Slovenia and other European countries and those available in the GenBank. The partial gC nucleotide sequences (663 nt) of the Slovenian PRV isolates showed 98.34 to 100% nucleotide identity among themselves. The partial gD nucleotide sequences (210 nt) of the Slovenian PRV isolates showed 100% nucleotide identity among themselves. The partial gE nucleotide sequences (453 nt) of the Slovenian PRV isolates showed 99.12 to 100% nucleotide identity among themselves. To determine the phylogenetic relationship between the five Slovenian PRV isolates from dogs and 38 PRV isolates from wild boars, domestic pigs, and dogs from other European countries and one PRV dog isolate from Asia used as an outgroup, a phylogenetic tree was constructed based on the comparison of 663 nt of the partial gC gene. According to the classification of Fonseca et al. [42], the Slovenian dog PRV strains belonged to clade A (Figure 4). The first Slovenian isolate JAN 05/06 (case No. 1/2006) had 100% nucleotide identity with strains isolated from dogs and domestic pigs in Italy (JQ768125, JQ786156, KP780805, and JQ768131), and 96.96 to 99.70% nucleotide identity with other isolates from Europe, as shown in Figure 4. The group of Slovenian PRV strains isolated from dogs in 2018–2020, with four identical strains 35776–1, 35776–2, 52727, and 1383 (cases No. 2/2018, No. 3/2018, No. 4/2019, and No. 5/2020), had 99.25% nucleotide identity with the closest PRV strains isolated from a domestic pig from Italy (JQ768114), domestic pig and wild boar from Croatia (KC865672 and KC865678), hunting dog from Germany (GQ862778), and domestic pigs from Greece (KT983810 and KT983811), and 95.78 to 98.94% nucleotide identity with other isolates from Europe as shown in Figure 4. A nucleotide comparison of the five Slovenian PRV isolates from dogs between 2006 and 2020 revealed 5 nucleotide substitutions C ⟶ T at position 53,188, C ⟶ T at position 53,476, C ⟶ G at position 53,485, C ⟶ T at position 53,486, C ⟶ G at position 53,662 and deletions of three nucleotides (CGA) at positions 53,090–53,092, and three nucleotides (GGC) at positions 53,133–53,135, corresponding to the Kaplan reference sequence (JF797218) (data not shown). To analyse the variability of amino acids between the five Slovenian PRV strains isolated from dogs, the partial gC gene amino acid sequences were aligned and compared. The alignment of 221 amino acid sequences revealed two amino acid differences between the strain from case No. 1/2006 and the group of four identical strains from cases No. 2/2018, No. 3/2018, No. 4/2019, and No. 5/2020. The first amino acid difference was detected at position 25 (deletion) and the second at position 35 (deletion) according to the Kaplan reference sequence JF797218. Eight substitutions A > V, E > K, P > L, P > S, T > A, V > L, E > D, and E > D were detected between the Slovenian sequence amino acid and the Kaplan reference sequence JF797218 at positions 57, 99, 153, 156, 179, 182, 183, and 185, respectively.

**Figure 5:**
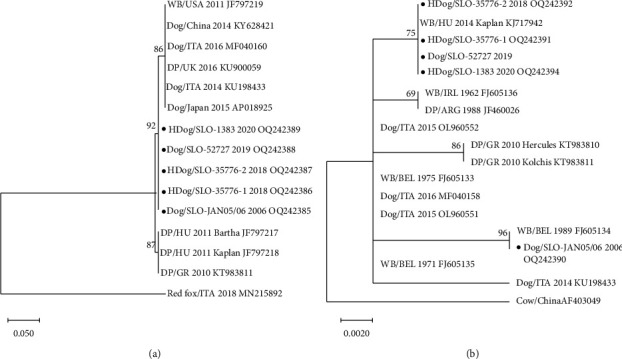
The phylogenetic comparison of the nucleotide sequences of partial gD (a) and gE (b) coding region of the five Slovenian PRV strains isolated from dogs and the 10 (a) or 13 (b) PRV sequences from wild boars, domestic pigs, red fox, and cows and dogs from Europe, China, Japan, and America. The tree was constructed by using the neighbour-joining method with *p*-distance and 1000 bootstrap replicates in MEGA version X [40]. For each strain species, country, the year of isolation, and accession number are reported (WB: wild boar, DP: domestic pig, and HDog: hunting dog). Slovenian PRV strains from this study are indicated with a black dot.

The comparison of the partial gD gene nucleotide sequences of the five Slovenian PRV isolates and 10 PRV isolates from the GenBank database (Figure 5) revealed that Slovenian PRV strains are 99.05% identical with PRV strains isolated from dogs (Italy: MF040160 and KU198433; Japan: AP018925; China: KY628421), domestic pigs (Hungary: F797217 and JF797218; Greece: KT983811), and wild boars (USA: JF797219). To analyse the variability of amino acids between the five Slovenian PRV strains isolated from dogs, the partial gD gene amino acid sequences were aligned and compared, and the alignment of 70 amino acid sequences revealed 100% identity between Slovenian PRV isolates.

The comparison of the partial gE gene nucleotide sequences of the five Slovenian PRV isolates and 13 PRV isolates from the GenBank database (Figure 5) revealed that Slovenian PRV strains are 99.12–100% identical with PRV strains isolated from dogs (Italy: MF040158, OL960552, and OL960551) and wild boars (Hungary: KJ717942; Belgium: FJ605133 and FJ605135). A nucleotide comparison of the five Slovenian PRV strains revealed 5 nucleotide substitutions between strain JAN 05/06 (case No. 1/2006) and a group of strains 35776–1, 35776–2, 52727, and 1383 (cases No. 2/2018, No. 3/2018, No. 4/2019, and No. 5/2020). To analyse the variability of amino acids between the five Slovenian PRV strains isolated from dogs, the partial gE gene amino acid sequences were aligned and compared. The alignment of the 151 amino acid sequences revealed 3 amino acid substitutions at positions 44 (V > L), 68 (P > T), and 95 (T > A).

## 4. Discussion

Since 2010, Slovenia has been free of PR in the domestic pig population [19], whereas PRV circulates in the wild boar population [20, 22]. In Slovenia, only seroprevalence studies for screening antibodies were performed to confirm the presence of PRV in the wild boar population. Because PRV circulates in wild boar populations, they pose a significant risk for PRV transmission between species, including dogs. Despite the high prevalence of seropositive wild boar in Slovenia, PRV cases in dogs, whether hunting dogs or pets, are rarely reported. Both hunting dogs and pet dogs can come in contact with potentially PRV-infected wild boars or their carcasses. However, PRV can also be imported with pigs or their parts from countries where it is also present in domestic pigs, as was suspected after an epidemiological investigation in case No. 1/2006. There was no information about the contact of the dog with the wild boar. The pig meat given to the dog was purchased in a butcher shop, and the pig was imported from Hungary, where PR was present at that time [43]. Most Slovenian PRV-positive cases came from hunting dogs (three cases No. 2/2018, No. 3/2018, and No. 5/2020) from the western part of the country, all of which participated in a wild boar hunt. The other two cases (No. 1/2006 and No. 4/2019) were from pet dogs fed with raw pork and wild boar meat, respectively. PRV infections occur sporadically, so only conspicuous cases are reported. Together with the study by Kotnik et al. [41], the present study is only the second report of PRV infection in dogs from Slovenia in over 30 years. All cases indicate direct interspecies transmission, similar to previously published results from other countries [10, 15, 23, 24, 26–33, 37], confirming the still existing risk of PR and supporting the need for national control programmes in the wild boar population for this disease.

The suspicion of PR in the Slovenian dogs was based on the typical clinical picture of severe, localized pruritus combined with information about the recent direct contact with wild boar or their carcasses or feeding raw wild boar or pork meat. The presence of uncontrollable facial pruritus, a pathognomonic sign of PR in nonnatural hosts was present in all Slovenian cases. According to Pejsak and Truszczynski [44], facial and neck pruritus associated with self-mutilation occurs in 17.8% to 52% of all PR cases described in dogs [16, 45] and is restricted to the face and neck area [15, 27, 31, 37]. In all described Slovenian PRV cases, infection occurred via the oral or respiratory mucosa, which was also confirmed by the anamnestic data. Detailed reports of pathologic lesions were available for only two of the five Slovenian PRV cases, No. 1/2006 and No. 5/2020. Because data on gross lesions were not available for the three cases (No. 2/2018, No. 3/2018, and No. 4/2019) and the first case (No. 1/2006) was further complicated by secondary bacterial infection, we cannot confirm whether and to what extent PRV infections in our dogs correspond to lesions observed in other studies [46].

Isolation of PR viruses on cell culture is not always successful [27], as was the case with the Slovenian samples, and depends on the course of the disease and the matrix used. In our study, the brain tissue was used as a matrix, which is the most common matrix [15, 26, 27]. After positive qPCR, successful virus isolation on cell culture, immunocytochemistry, PCR, and sequencing, phylogenetic analyses of the five Slovenian PRV isolates were performed on partial gC, gD, and gE genes. Within the phylogenetic analysis of the partial gC gene according to the classification of Fonseca et al. [42], five Slovenian PRV strains isolated from dogs were clustered into clade A, together with PRV sequences from dogs, wild boars, and domestic pigs from our neighbouring countries such as Croatia [28], Austria [29], Italy [26], and Hungary [6]. The phylogenetic analysis of the partial gD and gE genes of the five Slovenian PRV isolates revealed a similar conclusion as in the phylogenetic analysis of the partial gC gene where the Slovenian PRV strains are closely related with the PRV strains isolated from dogs, domestic pigs, and wild boars from neighbouring countries such as Italy [26] and Hungary [6].

When comparing the partial gC, gD, and gE gene sequences, Slovenian dog PRV strains show a low diversity among strains and a high genetic similarity to PRV strains detected in dogs, wild boars, and domestic pigs from Europe. The partial gC gene nucleotide sequence of the first Slovenian PRV isolate JAN 05/06 (case No. 1/2006) is 100% identical to the PRV strains from dogs and domestic pigs from the neighbouring country, Italy (JQ768125, JQ786156, KP780805, and JQ768131) from the period between 1993 and 2007. The partial gC, gD, and gE gene nucleotide sequences from the dog from case No. 1/2006, which was fed raw pork from Hungary, were closely related (97.98% for gC, 99.05% for gD, and 99.12% for gE) to PRV sequences from domestic pigs and wild boars from Hungary (GQ259113, GQ259114, F797217, JF797218, and KJ717942) isolated between 1997-1998 and 2011–2014. The results of our phylogenetic analysis indicate that PRVs similar to the Slovenian isolate JAN 05/06 (case No. 1/2006) were probably also present in Hungary in 2006.

Other four identical Slovenian PRV strains 35776–1, 35776–2, 52727, and 1383 (cases No. 2/2018, No. 3/2018, No. 4/2019, and No. 5/2020), found in a closely related geographical area near the Slovenian-Italian border, are closely related to the strains isolated from a domestic pig from Italy (JQ768114), domestic pig and wild boar from Croatia (KC865672 and KC865678), hunting dog from Germany (GQ862778), and domestic pigs from Greece (KT983810 and KT983811), with 99.25% nucleotide identity and 95.78 to 98.94% nucleotide identity with other isolates from Europe when comparing the partial gC gene. When comparing the partial gD and gE genes, Slovenian PRV strains 35776–1, 35776–2, 52727, and 1383 (cases No. 2/2018, No. 3/2018, No. 4/2019, and No. 5/2020) were closely related (99.05% identity for partial gD gene and 99.34–100% for partial gE gene) to the PRV strains from dogs from Italy (MF040160, KU198433, MF040158, OL960552, and OL960551) and domestic pigs and wild boars from Hungary (F797217, JF797218, and KJ717942). The characterization of the partial gC, gD, and gE genes of the PRV Slovenian isolates from dogs suggests that PRV strains were circulating in the Slovenian wild boar population in 2018–2020, as contact with wild boar or ingestion of raw wild boar meat or offal was confirmed in all the four cases (No. 2/2018, No. 3/2018, No. 4/2019, and No. 5/2020).

Analysis of the sequenced partial gC gene from the five Slovenian PRV isolates from dogs revealed the presence of two amino acid deletions, similar to strains isolated from dogs and domestic pigs from Italy (JQ768125, JQ786156, KP780805, and JQ768131). In Slovenia, the association between strains detected in domestic pigs and dogs and between wild boars and dogs could not be described, as was the case in Italy [26], because PRV sequences from pigs from Slovenia are not available. The domestic pig population in Slovenia does not pose a risk for PRV infection, as Slovenia is officially free of PR in the domestic pig population and no PRV outbreaks have been detected for more than two decades. In the Slovenian wild boar population, PRV infection is endemic, but no PRV has been isolated and characterized from wild boar, so phylogenetic comparisons can only point to the origin of PRV infection in Slovenian dogs. Because four detected cases (No 2/2018, No. 3/2018, No. 4/2019, and No. 5/2020) between 2018 and 2020 confirmed the contact with wild boar or raw wild boar meat, we could speculate that the PRV strain described here circulated in the Slovenian wild boar population between 2018 and 2020.

Genetic characterization of the PRV strains from the natural and nonnatural hosts is essential for a better understanding of the PRV diversity and for tracing the chain of infection to its origin [11]. The results of the genetic characterization of the partial gD and gE genes of the first case, No. 1/2006, show that the isolate was closely related to the isolates from Hungary isolated between 1997 and 1998 and 2011 and 2014 and suggest that it occurred in Hungary, where the pig whose meat was fed to the dog originated. The other four PRV cases, No. 2/2018, No. 3/2018, No. 4/2019, and No. 5/2020, are closely related to the first case when comparing the partial gC, gD, and gE genes and provide information on the strains detected in dogs after they were exposed to wild boar or wild boar meat during or after hunting. Because the gC, gD, and gE genes represent only a very limited part of the viral genome, further studies with analyses of the complete PRV genome are needed to draw conclusions about the diversity of circulating PRV, also in view of the fact that all five Slovenian isolates were not purified by the plaque assay, so the possibility that two or more PRV strains infected a dog cannot be excluded. In summary, the uncontrolled feeding of raw wild boar or pork meat or offal to domestic and hunting dogs poses a significant risk for the occurrence of PRV infection in this species. PRV is incurable in nonnatural hosts, so it is important to raise awareness among dog owners, hunters, wildlife biologists, and veterinarians. When a dog is infected with PRV, there is no treatment, and due to the severe course of the disease, euthanasia may be the most humane option.

## Figures and Tables

**Table 1:** PR cases reported between 2006 and 2020 from Slovenia and a summary of the main necropsy findings, histopathology of the brain and some other organs, and immunohistochemistry results for PRV in the brain of five dogs.

Case/year	Sample name	Species/breed	Age (years)/sex (M/F)	Sampling date	Municipality	Necropsy	Histopathology	IHC (brain)
No. 1/2006	Jan 05/06	Dog/crossbreed	2,5/F	January 3^rd^ 2006	Velike Lašče	Severe pulmonary oedema, multifocal myocardial haemorrhages, chronic nephritis and cystitis, chronic hyperplastic and acute haemorrhagic gastroenteritis, enlarged spleen, and enlarged lymph nodes	Severe nonpurulent meningoencephalitis and myelitis extending to cranial segments of the thoracic spinal cord, and severe pulmonary congestion and oedema. Severe acute necrotizing and haemorrhagic mix to cellular myocarditis. Chronic multifocal granulomatous nephritis	++++++ to +/− (†)
No 2/2018	35776-1	Hunting dog/NA	NA	November 6^th^ 2018	Koper	NA	Nonpurulent encephalomyelitis in the caudal brainstem (medulla). Pulmonary congestion, acute haemorrhages, severe pulmonary oedema, and atelectasis	+++
No 3/2018	35776-2	Hunting dog/NA	NA	November 6^th^ 2018	Koper	NA	Acute multifocal haemorrhages in the medulla and mild meningeal oedema. Pulmonary congestion, acute haemorrhages, severe pulmonary oedema, and atelectasis	++
No 4/2019	52727	Dog/Border Collie	5/M	December 27^th^ 2019	Nova Gorica	General congestion and oedema	Circumscribed nonpurulent encephalomyelitis in the caudal brainstem (medulla), neuronal necrosis in the cerebral cortex, brain stem, and the cerebellum. Severe pulmonary congestion, oedema, acute haemorrhages, and multifocal vasculitis of the lung. Pustular and ulcerative dermatitis of the nose. Chronic gastroenteritis	++
No 5/2020	1383	Hunting dog/Brandl Bracke	NA/F	January 16^th^ 2020	Koper	Generalised congestion, oedema of the cutis and subcutis, strong pharyngeal and laryngeal oedema, alopecia of the facial skin, strong splenic hyperaemia, and enlarged lymph nodes	Meningeal oedema and hyperaemia, a small number of disseminated lymphocytes and histiocytes in the leptomeninges, and acute perivascular haemorrhages in the medulla. Severe pulmonary congestion, oedema, and atelectasis	±

(†) IHC for the thoracic spinal cord is included beside the brain; positivity was strong in proximal segments and disappeared in distal. NA, not applicable.

**Table 2:** Primers used in the PCRs.

Primer	Primer sequence (5′ 3′)	Glycoprotein	Expected product (b.p.)	Reference
gC-For	GTG CGC CAC TAG CAT TAA ATC CGT	gC	800	[6]
gC-Rev	CTG TAC AGG AGG AGC GAG ACG TT	gC	800	[6]
FgDm	GTG CAC GGA GGA CGA GCT GGG GCT	gD	217	[37]
RgDm	GAC GTC CAC GCC CCG CTT GAA GCT	gD	217	[37]
gE-nF	CCG CGG GCC GTG TTC TTT GT	gE	493	[38]
gE-nR	CGT GGC CGT TGT GGG TCA T	gE	493	[38]

**Table 3:** The detected *Cq* values by specific PRV real-time PCR from the original samples of tissue homogenate and from suspensions of PRV isolates from cell culture of PK-15 (3 passages) and virus titer.

Case/year/sample name	*Cq* value in the original samples of tissue homogenate		*Cq* value in suspensions of PRV isolate from cell culture of PK-15 (1^st^ passage)		*Cq* value in suspensions of PRV isolate from cell culture of PK-15 (2^nd^ passage)		*Cq* value in suspensions of PRV isolate from cell culture of PK-15 (3^rd^ passage)		Virus titer
	gE region	gB region	gE region	gB region	gE region	gB region	gE region	gB region	
No. 1/2006/Jan 05/06	ND	ND	19.19	18.15	16.22	16.31	15.60	15.38	5.6 × 10^8^/ml
No. 2/2018/35776-1	29.33	29.34	24.35	24.03	21.33	21.54	16.89	17.55	5.6 × 10^8^/ml
No. 3/2018/35776-2	33.91	34.59	18.66	19.01	16.53	16.94	15.18	15.09	4.2 × 10^8^/ml
No. 4/2019/52727	33.91	33.72	16.70	16.61	15.80	15.20	14.12	14.53	1.3 × 10^8^/ml
No. 5/2020/1383	33.42	32.61	14.97	14.81	13.52	12.85	11.29	11.77	3.1 × 10^7^/ml

ND, not done.

## Data Availability

The nucleotide sequence data used to support the findings of this study have been deposited in the GenBank repository (ON025041-45 and OQ242385-94).
